# Identification of differential microRNAs and messenger RNAs resulting from ASXL transcriptional regulator 3 knockdown during during heart development

**DOI:** 10.1080/21655979.2022.2062525

**Published:** 2022-04-17

**Authors:** Ze-Qun Liu, Mi Cheng, Fang Fu, Ru Li, Jin Han, Xin Yang, Qiong Deng, Lu-Shan Li, Ting-Ying Lei, Dong-Zhi Li, Can Liao

**Affiliations:** a Department of Prenatal Diagnostic Center, Guangzhou Women and Children’s Medical Center, Guangzhou Medical University, Guangzhou Guangdong, China; b Department of Obstetrics, Guangzhou Women and Children’s Medical Center, Guangzhou Medical University, Guangzhou Guangdong, China

**Keywords:** ASXL transcriptional regulator 3, heart development, microRNA, messenger RNA, PI3K-akt

## Abstract

Congenital heart disease (CHD) is the most common birth defect. Although ASXL transcriptional regulator 3 (ASXL3) has been reported to cause hereditary CHD, ASXL3-mediated mechanisms in heart development remain unclear. In this study, we used dimethyl sulfoxide (DMSO) to induce differentiation in P19 cells, observed cell morphology using light microscopy after ASXL3 knockdown, and determined the levels of associated myocardial cell markers using reverse transcription-quantitative polymerase chain reaction and western blotting. Subsequently, we used microRNA sequencing, messenger RNA (mRNA) sequencing, and bioinformatics to initially identify the possible mechanisms through which ASXL3-related microRNAs and mRNAs affect heart development. The results indicated that DMSO induced P19 cell differentiation, which could be inhibited by ASXL3 knockdown. We screened 1214 and 1652 differentially expressed microRNAs and mRNAs, respectively, through ASXL3 knockdown and sequencing; these differentially expressed miRNAs were largely enriched in PI3K-Akt, mitogen-activated protein kinase, and Rap1 signaling pathways. Additionally, 11 miRNAs associated with heart development were selected through a literature review. Our analysis indicated the involvement of mmu-miR-323-3p in P19 cell differentiation through the PI3K-Akt pathway. In conclusion, ASXL3 may be involved in the regulation of heart development. This comprehensive study of differentially expressed microRNAs and mRNAs through ASXL3 knockdown in P19 cells provides new insights that may aid the prevention and treatment of CHD.

## Graphical abstract

**Figure:**
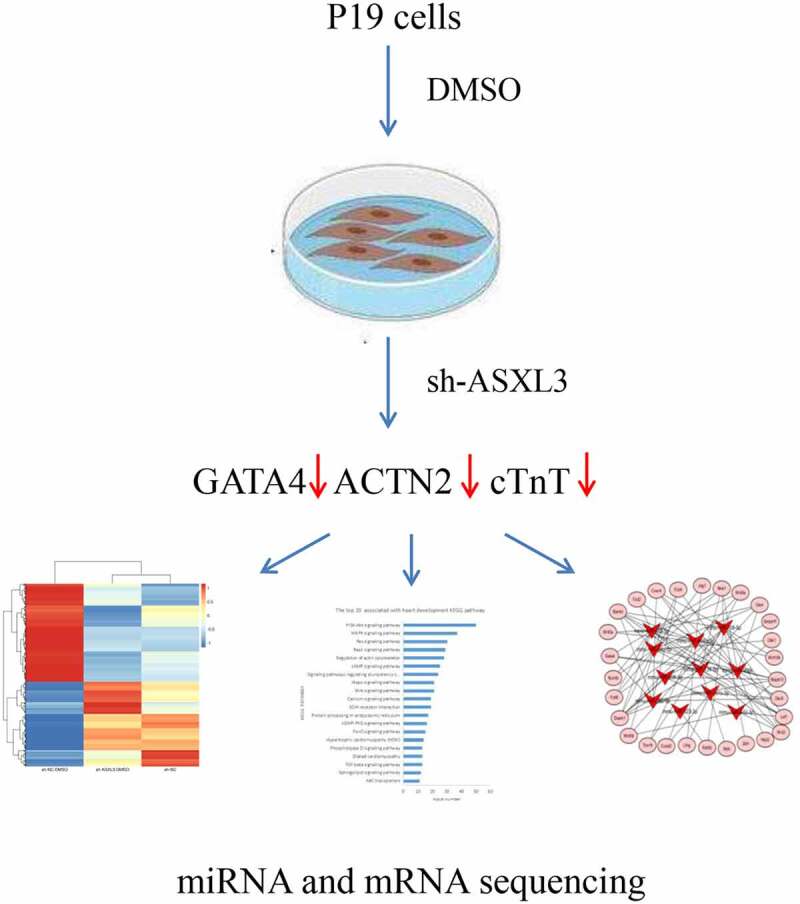

## 1. Introduction

Congenital heart disease (CHD) refers to malformations due to the aberrant development of the heart and major vessels, such as heart wall, heart valve, and vascular malformations, during the embryonic stage[1]. CHD is the leading cause of noninfectious death in infants, accounting for one-third of all major congenital malformations [2]. Additionally, the current CHD prevalence ranges from 0.8% to 1.2% [3]. A systematic review published in 2019 investigated the global incidence of neonatal CHD from 1970 to 2017 [4]. Ventricular and atrial septal defects reportedly have the highest incidence rates [5]. CHD has high morbidity and mortality rates in adult mammals because of the lack of regenerative capacity [6]. Owing to the complex pathophysiology of CHD, only 20% of CHD-related genes have been identified lately, thus greatly limiting the advancement of clinical therapy for CHD [7]. Therefore, research on this topic must be expedited.

Mutated genes associated with CHD are considered to be involved primarily in essential regulatory functions in the early phase of heart development; these regulatory factors include cardiac transcription factors, heart-specific genes, or signaling pathway molecules [8,9]. ASXL transcriptional regulator gene (ASXL) is the homologous gene of Drosophila sp. additional sex comb-like gene in humans and has three different subtypes: ASXL1, ASXL2, and ASXL3 [10]. ASXL is an epigenetic regulatory factor that contributes to the development of polycomb-group and trithorax-group complexes, which have transcriptional regulatory functions [11]. Previous studies have elucidated that embryogenic mutations in ASXL1, ASXL2, and ASXL3 may lead to Bohring–Opitz syndrome [12], Shashi–Pena syndrome [13], and Bainbridge–Ropers syndrome [14,15], respectively. Furthermore, ASXL3 mutations have been detected in individuals with autism [16] or extreme short stature [17]. Heterozygous missense mutations of ASXL3 C.2168C>G (p.P723R) and C.5449C>G (p.P1817A) reportedly cause autosomal recessive CHD [18]. In addition, ASXL3 mutation can cause varied splicing of several genes in ASXL3 (P723R/ P1817A) mice with complex heterozygous mutation [19]. Therefore, ASXL3 may be involved in the regulation of heart development.

Heart development involves the precise orchestration of gene expression during heart differentiation and morphogenesis by evolutionarily conserved regulatory networks. In the cardiovascular system, microRNAs (miRNAs) play their physiological and pathological roles in heart development [20,21] and disease [22,23]. A recent study has reported that a series of miRNAs, including hsa‐miR‐590 and hsa‐miR‐199a, promote mammalian cardiomyocyte proliferation by activating the nuclear translocation of Yes1 associated transcriptional regulator (YAP) and inducing the expression of YAP-responsive genes [24,25]. Another study found that mice lacking either miR-133a-1 or miR-133a-2 were normal, whereas the deletion of both miRNAs caused lethal ventricular septal defects in approximately half of double-mutant embryos or neonates [26]. ASXL3 mutation substantially modify the expression profiles of long noncoding RNA and messenger RNA (mRNA) in the mouse cerebrum and cerebellum, and ASXL3 knockdown via small interfering RNA transfection affects the proliferation, cell cycle progression, and apoptosis of neural cells [27]. However, knowledge regarding the effects of ASXL3 knockdown on cardiomyocyte miRNAs and mRNAs remains limited.

In this study, we supposed that ASXL3 may affect miRNAs and mRNAs expression in cardiomyocyte. To test our speculation, dimethyl sulfoxide (DMSO) was used to induce the differentiation of P19 cells into cardiomyocytes. ASXL3 interference influenced this DMSO-mediated induction effect. Subsequently, we performed miRNA and mRNA sequencing and bioinformatic analysis to screen out candidate differential genes associated with ASXL3 in P19 cell-derived cardiomyocytes, which will help the experimental biologists and clinicians to further carry forward outcomes to treat patients like previous studies [28–31]. Therefore, this study may provide a scientific basis for better understanding CHD and relevant clinical therapy.

## 2. Materials and methods

### 2.1 Cellular induction and differentiation

P19 cells were acquired from American Type Culture Collection (Manassas, VA, USA; Cat. No. CRL-1825) and cultured at 37°C under 5% CO_2_ in alpha-minimum essential medium (α-MEM; Invitrogen, Carlsbad, CA, USA) with 10% fetal bovine serum (Gibco, Waltham, MA, USA). As described in previous studies [32], P19 cells were stimulated with 1% DMSO (Sigma, D4540) for 4 days to induce differentiation. Then, 30–40 embryo-like bodies were inoculated into a six-well plate with α-MEM for further culture. The medium was replaced every 2 days. The morphological changes in P19 cells were observed under an inverted microscope (CNOPTEC, Chongqing, China).

### 2.2 Ribonucleic acid (RNA) interference

The sh-ASXL3 interference lentiviral vector and its negative control vector (sh-NC) were obtained from GenePharma (Shanghai, China). The sh-ASXL3 sequences were as follows: 5ʹ-GCTGAAGGCATTTGCATTA-3ʹ and 5ʹTAATGCAAATGCCTTCAGC-3ʹ. DMSO-treated and untreated P19 cells (1 × 10^5^ cells/well) seeded onto six-well plates were stably infected with sh-NC or sh-ASXL3. Then, the infected cells were observed using fluorescence microscopy, and ASXL3 interference efficiency was verified using reverse transcription-quantitative polymerase chain reaction (RT-qPCR) and western blotting.

### 2.3 Plasmid construction

For plasmid construction, empty control and pcDNA3.1-ASXL3 vectors were transfected into P19 cells using Lipofectamine 2000 (Thermo Fisher, USA) according to the manufacturer’s instructions.

### 2.4 Real-time RT-qPCR

TRIzol (Beyotime, Shanghai, China) was used to extract total RNA from processed P19 cells. Reverse transcription was performed using the First Strand cDNA Synthesis Kit (TaKaRa Bio Inc., Shiga, Japan). SYBR Green qPCR SuperMix (Invitrogen, Carlsbad, CA, USA) was used for gene amplification. The results were interpreted using the 2^−ΔΔCT^ method [33]. The primers were synthesized by Generay Biotech (Generay, Shanghai, China). The premier sequences were as follows:

ASXL3: 5ʹ-CCCTATGACCAGAACGAAGTGA-3ʹ (Forward)

5ʹ-CCCAAAGTGTATCGTCGGGTAA-3ʹ (Reverse)

GATA binding protein 4 (GATA4): 5ʹ-GGGTAGCCCTGGCTGGA-3ʹ (Forward)

5ʹ-GGTAGGGGCTGGAGTAGGAG-3ʹ (Reverse)

Cardiac troponin T (cTnT): 5ʹ-TGCCTGCTTAAAGCTCTCCC-3ʹ (Forward)

5ʹ-CTCTCGGCTCTCCCTCTGAA-3ʹ (Reverse)

Actinin alpha 2 (ACTN2): 5ʹ-CTTCTACCATGCTTTCGCGG-3ʹ (Forward)

5ʹ-GGGCTTATGCTTACGACGGT-3ʹ (Reverse)

### 2.5 Western blotting

The processed P19 cells were washed with and dissolved in phosphate buffer saline (Gibco, Carlsbad, CA, USA) and homogenized on ice for 40 min in cold radioimmunoprecipitation assay buffer with phosphatase inhibitors. Total protein was extracted using high-speed centrifugation. Protein (50 μg) was separated via 10% sodium dodecyl sulfate–polyacrylamide gel electrophoresis and transferred onto polyvinylidene fluoride membranes. After blocking, the membranes containing the target protein were incubated with primary antibodies (1:1000) at 4°C overnight and horseradish peroxidase-conjugated secondary antibodies (1:1000) for 2 h. The gray value was obtained using the ImageJ 2 software after visualizing the interaction with ECL reagents. The primary antibodies used were anti-ACTN2 (ProteinTech, Wuhan, China), anti-GATA4 (ProteinTech), anti-ASXL3 (GenScript Biotech, Nanjing, China), and anti-cTnT (ProteinTech).

## 2.6 mRNA sequencing

Total RNA was extracted from cells using TRIzol (Invitrogen) according to the manufacturer’s instructions. The RNA quality and integrity of each sample were initially tested using the 2100 bioanalyzer (Agilent Technologies) and NanoDrop spectrophotometer. Then, a cDNA library was constructed. mRNA containing poly-a tail was purified using poly-T magnetic beads, and the purified mRNA was randomly split into 300-bp fragments. The DNA fragment was enriched through PCR after reverse complementation, and ‘A’ was appended to its 3ʹ end. The HiSeq 4000 (Illumina ®, SanDiego, CA, USA) platform was used to sequence cDNA libraries [34].

## 2.7 miRNA sequencing

Total RNA was extracted from cells and prepared using a mirVana miRNA Isolation Kit (Invitrogen) according to the manufacturer’s instructions. Similar to the principle of mRNA sequencing, cDNA libraries were first constructed using TruSeq® Small RNA Sample Prep Kit (Illumina, San Diego, CA, USA). The cDNA libraries were then sequenced using the HiSeq 2000 (Illumina, San Diego, CA, USA) platform as described previously [35].

### 2.8 Gene ontology analysis

The results of miRNA and mRNA sequencing indicated that differentially expressed miRNAs (DE-miRNAs) and differentially expressed mRNAs (DEMs) were screened out under the screening criteria of p ≤ 0.05 and |log2 ratio | ≥ 0.59. As described in previous studies [36,37], gene ontology (GO) analysis was used to map these differentially expressed genes to each term in the GO database (http://www.geneontology.org) and calculate the number of genes associated with each term. A hypergeometric test was then used to determine the distribution of target genes enriched in each term compared with all background genes.

### 2.9 Kyoto encyclopedia of genes and genomes analysis

Using the same screening criteria described in section 2.8, differentially expressed miRNAs and mRNAs were screened out. And the Kyoto Encyclopedia of Genes and Genomes (KEGG) database was used to analyze the metabolic pathway enrichment of differentially expressed genes. The metabolic and/or signal transduction pathways that were significantly enriched with these genes were identified through a comparison with the genome-wide background, and a hypergeometric test was used to determine the significantly enriched metabolic pathways. To identify the significant enrichment of pathways, pathway enrichment analysis was also performed based on the KEGG database and differentially expressed genes. The KOBAS software was used to confirm the significant enrichment of the KEGG pathway with the target genes, as described in a previous study [38].

### 2.10 Statistical analysis

The data were expressed as means ± standard deviations and analyzed using SPSS (version 23.0, Chicago, IL, USA) with one-way analysis of variance or Student’s t-test. Duncan’s multiple range test was used for the correlation analysis of miRNA and mRNA between samples. Statistical significance was set at p < 0.05.

## 3. Results

In this study, the differentiation of P19 cells was induced by DMSO, and the role of ASXL3 in cardiac development was investigated through ASXL3 knockdown or overexpression. Overall, we demonstrated that ASXL3 may be involved in the regulation of cardiac development. Based on the different expression profiles of miRNA and mRNA resulting from ASXL3 knockdown, we used bioinformatics to identify the most promising ASXL3–miRNA–mRNA networks associated with the PI3K-Akt and mitogen-activated protein kinase (MAPK) signaling pathways during heart development.

### 3.1 ASXL3 was upregulated during DMSO-induced P19 cell differentiation

**Figure 1.**
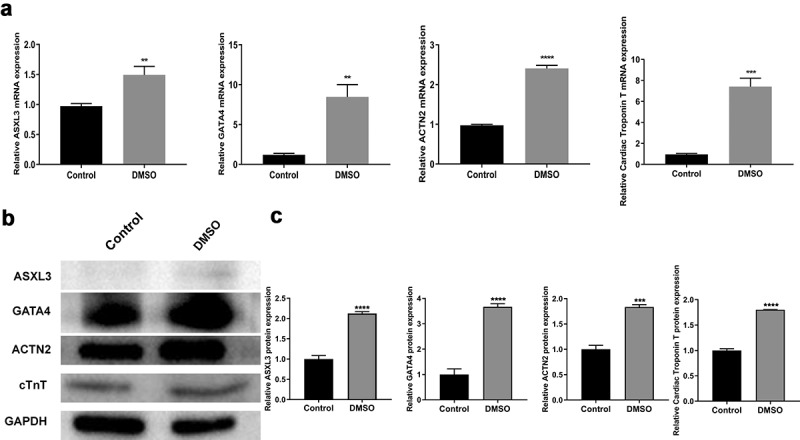
ASXL transcriptional regulator 3 (ASXL3) was upregulated in dimethyl sulfoxide (DMSO)-induced differentiation of P19 cells. P19 cells were treated with 1% DMSO to induce differentiation. (a) Reverse transcription-quantitative polymerase chain reaction was performed to assess the expression of ASXL3, GATA binding protein 4 (GATA4), actinin alpha 2 (ACTN2), and cardiac troponin T (cTnT) in DMSO-treated P19 cells. (b) Western blotting was performed to assess the expression of ASXL3, GATA4, ACTN2, and cTnT in DMSO-treated P19 cells, and the expression levels were quantified based on western blotting results. **p < 0.01, ***p < 0.001, and ****p < 0.0001.

To study the regulatory mechanism of heart development, we induced P19 cell differentiation using 1% DMSO. Interestingly, ASXL3 expression was significantly higher in the DMSO group than in the control group, and cardiomyocyte marker genes (GATA4, ACTN2, and cTnT) were also upregulated in the DMSO group compared with the control group (Figure 1a). Similarly, western blotting results indicated that the expression of ASXL3, GATA4, ACTN2, and cTnT proteins was higher in the DMSO group than in the control group (Figure 1b). Overall, it can be considered that the myocardial differentiation model of P19 cells was successfully constructed and ASXL3 was highly expressed in this cell model.

### 3.2 ASXL3 was steadily knocked down in P19 cells

**Figure 2.**
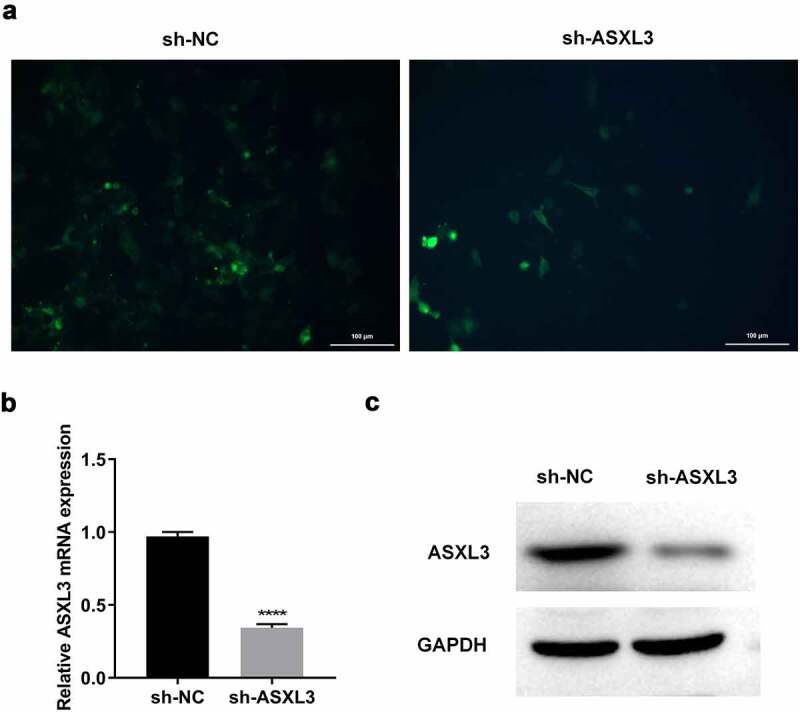
ASXL transcriptional regulator 3 (ASXL3) was steadily knocked down in P19 cells. P19 cells were stably infected with sh-ASXL3. (a) Fluorescence of the infected P19 cells was observed using a fluorescence microscope. ASXL3 expression was monitored via reverse transcription-quantitative polymerase chain reaction (b) and western blotting (c). ***p < 0.001 and ****p < 0.0001.

To further investigate the effects of ASXL3 knockdown on DMSO-induced P19 cells, we first knocked down ASXL3 in P19 cells via transfection. High fluorescence intensity (Figure 2a) indicated that transfection was successful. RT-qPCR results suggested that the mRNA level of ASXL3 was significantly reduced in the sh-ASXL3 group compared with the sh-NC group (Figure 2b and Supplemental Figure 1). Furthermore, sh-ASXL3 transfection significantly reduced the ASXL3 level in P19 cells (Figure 2c). Overall, the results suggest that sh-ASXL3 was successfully introduced into P19 cells.

### 3.3 ASXL3 knockdown suppressed DMSO-induced P19 cell differentiation

**Figure 3.**
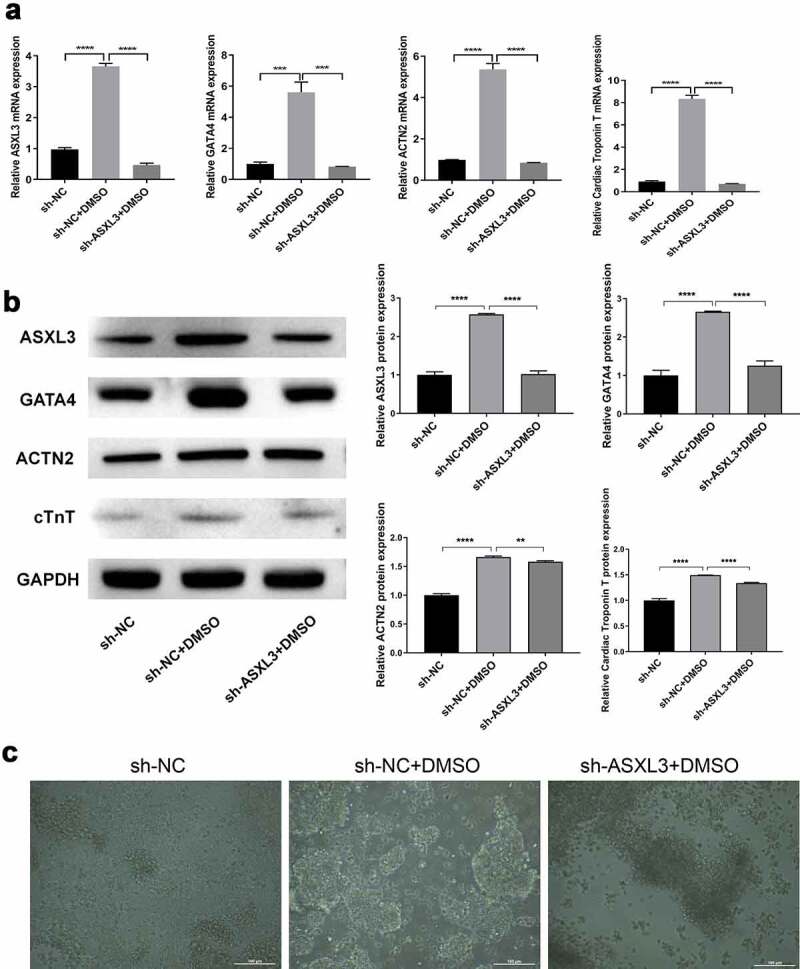
ASXL transcriptional regulator 3 (ASXL3) knockdown suppressed dimethyl sulfoxide (DMSO)-induced P19 cell differentiation. P19 cells with ASXL3 knockdown were treated with 1% DMSO for 4 days. (a) Reverse transcription-quantitative polymerase chain reaction revealed the changes in the expression profiles of GATA binding protein 4 (GATA4), actinin alpha 2 (ACTN2), and cardiac troponin T (cTnT). (b) Protein expression by the above mentioned four genes was evaluated using western blotting. (c) Morphology of P19 cells was observed using a light microscope at the end of induction for 4 days. **p < 0.01, ***p < 0.001, and ****p < 0.0001.

We further verified whether ASXL3 affects P19 cell differentiation. As shown in Figure 3a, the expression of ASXL3, GATA4, ACTN2, and cTnT was noticeably higher in the sh-NC + DMSO group than in the sh-NC group; however, the expression of these four genes was evidently lower in the sh-ASXL3 + DMSO group than in the sh-NC + DMSO group. Similarly, western blotting results indicated that ASXL3 knockdown significantly inhibited the upregulation of ASXL3, GATA4, ACTN2, and cTnT in DMSO-treated P19 cells (Figure 3b). The P19 cells in the sh-NC group were noted to be uniform in size and small. In the DMSO induction group, embryonic-like bodies were formed and spindle cells increased. ASXL3 interference suppressed DMSO-induced P19 cell differentiation (Figure 3c). Consequently, ASXL3 knockdown may play a remarkable inhibitory role in DMSO-induced differentiation of P19 cells.

### 3.4 ASXL3 overexpression rescued sh-ASXL3-mediated inhibition of DMSO-induced P19 cell differentiation

**Figure 4.**
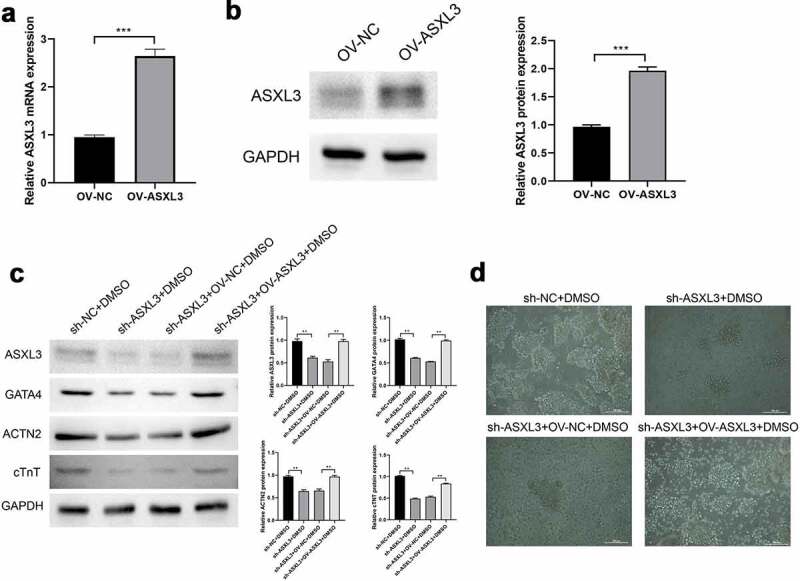
ASXL transcriptional regulator 3 (ASXL3) overexpression rescued sh-ASXL3-mediated inhibition of dimethyl sulfoxide (DMSO)-induced P19 cell differentiation. After DMSO was used to induce P19 sh-ASXL3 cells, pcDNA3.1-ASXL3 was used to transfect the cells. (a) Reverse transcription-quantitative polymerase chain reaction revealed the expression of ASXL3. (b) Protein expression of ASXL3 was evaluated using western blotting. (c) Protein expression of ASXL3, GATA binding protein 4 (GATA4), actinin alpha 2 (ACTN2), and cardiac troponin T (cTnT) was evaluated using western blotting. (d) P19 cell morphology was observed using a light microscope. **p < 0.01 and ***p < 0.001.

We verified the role of ASXL3 in P19 cell differentiation by overexpression of ASXL3. As shown in Figures 4Aandb, ASXL3 mRNA and protein were significantly upregulated by overexpressed ASXL3. The expression of ASXL3, GATA4, ACTN2, and cTnT proteins was noticeably lower in the sh-ASXL3 + DMSO group than in the sh-NC + DMSO group; ASXL3 overexpression significantly reversed the above mentioned effects (Figure 4c). Furthermore, in the DMSO induction group, embryonic-like bodies were formed and the number of spindle cells increased; however, ASXL3 interference suppressed DMSO-induced differentiation of P19 cells, whereas ASXL3 overexpression significantly reversed these effects (Figure 4d). Therefore, ASXL3 overexpression may play an important role in promoting DMSO-induced differentiation of P19 cells.

### 3.5 Identification of DE-miRNAs in DMSO-induced P19 cells after ASXL3 knockdown

**Figure 5.**
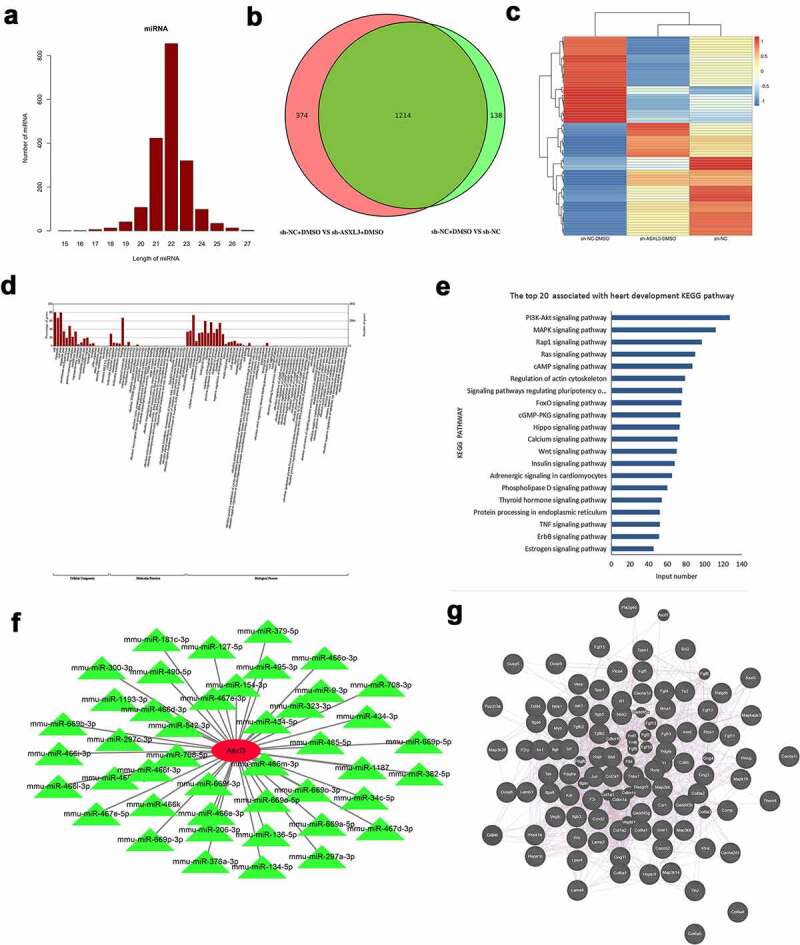
Identification of differentially expressed microRNAs (DE-miRNAs) in dimethyl sulfoxide (DMSO)-induced P19 cells after ASXL transcriptional regulator 3 (ASXL3) knockdown. After ASXL3 knockdown, miRNA sequencing was performed to confirm the changes in the miRNA profile of DMSO-treated P19 cells. (a) Length distribution of the screened miRNAs. (b) Venn diagram of common DE-miRNAs (p ≤ 0.05 and |log2Ratio| ≥ 0.59). (c) Heat map of DE-miRNAs between the three groups. (d) Gene ontology analysis of 1214 DE-miRNAs. (e) Kyoto Encyclopedia of Genes and Genomes analysis of the 1214 DE-miRNAs. (f) Network diagram of common DE-miRNAs associated with ASXL3. (g) Interacting partners of ASXL3 in the PI3K-Akt, mitogen-activated protein kinase (MAPK), and Rap1 signaling pathways.

To further investigate the aberrant miRNAs associated with ASXL3 during heart development, we performed miRNA sequencing to screen out DE-miRNAs after ASXL3 knockdown in DMSO-induced P19 cells. Based on the sequencing data, we found that the length of the screened miRNAs varied mainly from 21 to 23 nt (Figure 5a). A Venn diagram revealed that there were 1214 common DE-miRNAs (sh-NC + DMSO vs. sh-ASXL3 + DMSO and sh-NC + DMSO vs. sh-NC), with 55 upregulated miRNAs and 1159 downregulated miRNAs based on p ≤ 0.05 and |log2Ratio| ≥ 0.59 (Figure 5b and Table S1). A heat map was used to show the distribution of DE-miRNAs (Figure 5c). In addition, GO analysis revealed that 1214 DE-miRNAs were enriched primarily in cellular components and biological processes (Figure 5d and Table S2). KEGG analysis revealed that 1214 differential miRNAs were mostly enriched in the top three signaling pathways, PI3K-Akt (n = 127), MAPK (n = 112), and Rap1 (n = 97; Figure 5e and Table S3). Furthermore, we found 44 common DE-miRNAs associated with ASXL3 (figure 5f and Table S4); of these, 11 miRNAs were associated with heart development, as evident through a literature review: mmu-miR-206-3p, mmu-miR-181c-3p, mmu-miR-708-5p, mmu-miR-485-5p, mmu-miR-323-3p, mmu-miR-382-5p, mmu-miR-134-5p, mmu-miR-466k, mmu-mir-34c-5p, mmu-miR-542-3p, and mmu-miR-466d-3p (Table S5 and S6). Given that differentially expressed genes were mainly enriched in the PI3K-Akt, MAPK, and Rap1 pathways, we explored the interaction between ASXL3 and molecules related to these pathways using the GeneMANIA (http://genemania.org/) online prediction tool; using this tool, we predicted the correlation between ASXL3 and relevant pathway-related genes (Figure 5g). These findings showed that these 11 miRNAs were differentially expressed because of ASXL3 knockdown during heart development.

### 3.6 Identification of DEMs in DMSO-induced P19 cells after ASXL3 knockdown

**Figure 6.**
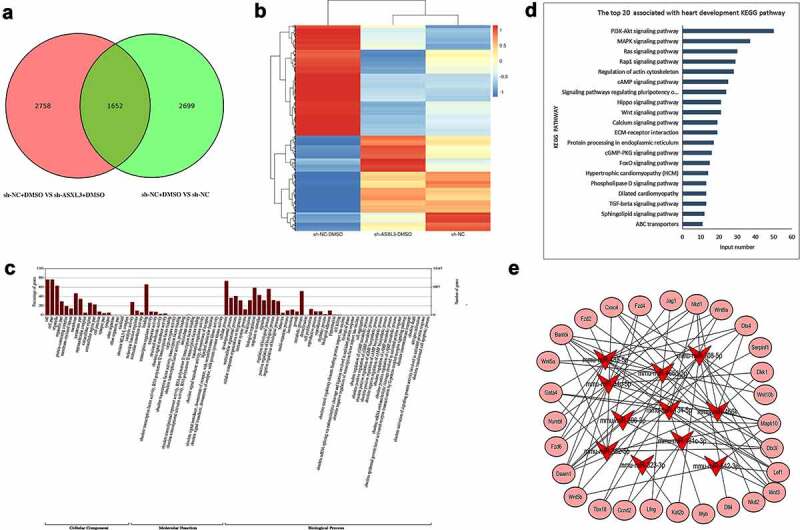
**Identification of differentially expressed messenger RNAs (DEMs) in dimethyl sulfoxide (DMSO)-induced P19 cells after ASXL transcriptional regulator 3 (ASXL3) knockdown**. mRNA sequencing was performed to assess the changes in the mRNA profile of DMSO-induced P19 cells after ASXL3 interference. (a) Venn diagram of common differentially expressed genes (p ≤ 0.05 and |log2Ratio| ≥ 0.59). (b) Heat map of DEMs among sh-NC, sh-NC + DMSO, and sh-ASXL3 + DMSO groups. (c) Gene ontology analysis of 1652 DEMs. (d) Kyoto Encyclopedia of Genes and Genomes analysis of the 1652 DEMs. (e) Network diagram of common DEMs associated with the screened 11 miRNAs involved in heart development.

We performed mRNA sequencing to preliminarily confirm the changes in mRNA expression in DMSO-treated P19 cells after ASXL3 interference. First, a Venn diagram revealed 1652 common DEMs between sh-NC + DMSO versus sh-ASXL3 + DMSO and sh-NC + DMSO versus sh-NC (p ≤ 0.05 and |log2Ratio| ≥ 0.59) groups, of which 941 were upregulated and 711 were downregulated in the ASXL3 knockdown group (Figure 6a and Table S7). The heat map represents the distribution of common DEMs (Figure 6b). Second, GO analysis revealed that 1652 common DEMs were enriched mainly in cellular components and biological processes (Figure 6c and Table S8). Moreover, KEGG analysis revealed that 1652 common DEMs were enriched in the top three signaling pathways: PI3K-Akt (n = 50), MAPK (n = 37), and Rap1 (n = 30; Figure 6d and Table S9). Finally, through bioinformatics, we identified mRNAs associated with heart development in the common DEMs and used these mRNAs for targeted analysis with the 11 miRNAs mentioned above (Figure 6e and Table S10).

### 3.7 Combined miRNA and mRNA analysis results

The results of a literature review of the 11 miRNAs are presented as supplementary material (Table S10). Of these 11 miRNAs, 6 showed consistent patterns in the literature (Table S11). KEGG analysis revealed mRNAs with a potential association with these 11 miRNAs and that participate in the PI3K-Akt, MAPK, and Rap1 signaling pathways: mmu-miR-134-5p-Myb, mmu-miR-181c-3p-Ccnd2, and mmu-miR-323-3p-Ccnd2 in the PI3K-Akt pathway and mmu-miR-206-3p-Mapk10, mmu-miR-34c-5p-Mapk10, mmu-miR-382-5p-Mapk10, mmu-miR-466k-Mapk10, mmu-miR-542-3p-Mapk10, and mmu-miR-708-5p-Mapk10 in the MAPK pathway (Table S12). After further analysis, we noted that mmu-miR-206-3p, mmu-miR-181c-3p, mmu-miR-708-5p, and mmu-miR-323-3p were all present in the abovementioned two tables and the predicted target genes were enriched in the PI3K-Akt and MAPK signaling pathways. In summary, these data verified our preliminary screening of ASXL3 knockdown-mediated DEMs, which were associated with the four miRNAs in DMSO-induced P19 cells via the PI3K-Akt and MAPK signaling pathways.

## 4. Discussion

The heart is the first organ formed during embryonic development [39]. Heart development involves multiple gene expression, cell differentiation, migration, transformation, and proliferation [40]. The precise regulation of gene expression ensures normal heart formation, and any disorder due to genetic or environmental factors can result in CHD [9]. CHD can cause delayed brain development, decreased physical endurance, pulmonary hypertension, arrhythmias, heart failure, and even death due to abnormalities in heart structure and hemodynamics [41–43]. ASXL3 belongs to the ASXL family and is located at 18ql2.1 [44]. It is an enhancer of the Polycomb and Trithorax families and contributes to embryonic development, cell proliferation, and tumor formation through transcriptional regulation [45]. In addition, ASXL3 is abundantly expressed in the testis, ovary, and brain tissues [46,47], indicating that ASXL3 plays a crucial role in embryonic heart development. Recent evidence suggests that ASXL3 mutations lead to autosomal recessive CHD [18]. In the present study, we first induced P19 cell differentiation using 1% DMSO, which is similar to the methods used for constructing cardiomyocyte differentiation model cells in previous studies [48,49]. Our results indicated that after DMSO stimulation, the expression of cardiomyocyte marker genes (GATA4, ACTN2, and cTnT) increased in P19 cells, suggesting that the myocardial differentiation model of P19 cells was successfully constructed. We further demonstrated that ASXL3 was upregulated in differentiated P19 cells and ASXL3 knockdown inhibited DMSO-induced differentiation of P19 cells; however, ASXL3 overexpression significantly promoted DMSO-induced differentiation of P19 cells, indicating that changes in the expression of ASXL3 are associated with heart development.

The formation of the heart is extremely complex. The complexity of cellular machinery depends more on the regulation of genes than on the number of genes [50]. With advances in the sequencing technology, large segments of the human genome, although efficiently transcribed, were detected to not encode proteins; these segments are called noncoding RNAs (ncRNAs) [51]. miRNAs – a type of short ncRNA – regulate gene expression at the post transcriptional level [52,53]. miRNAs are highly conserved in evolution and have stringent spatial and tissue specificities [22]. These RNAs affect the expression of multiple target genes, and different miRNAs can bind to the same target gene, resulting in an extremely large, complex, and fine regulatory network [54]. Recent studies have identified a close relationship between heart development and multiple miRNAs such as miR-294 [55], miR-430a [56], miR-29b-3p [57], and miR-182 [58]. Therefore, miRNAs may play a crucial role in heart development. In the present study, miRNA sequencing identified 1214 common DE-miRNAs in P19 cells treated with DMSO and sh-ASXL3. These differential miRNAs were enriched mainly in cellular components and biological processes as well as three signaling pathways (PI3K-Akt, MAPK, and Rap1). Furthermore, 11 ASXL3-mediated miRNAs were detected, which are associated with heart development (Results section).

mRNA sequencing further identified 1652 common DEMs in P19 cells treated with DMSO and sh-ASXL3. These differential mRNAs resulting from ASXL3 knockdown were enriched mainly in cellular components and biological processes as well as three signaling pathways (PI3K-Akt, MAPK, and Rap1). Moreover, through bioinformatics analysis, we identified three ASXL3–miRNA–mRNA networks (ASXL3-miR-134-5p-MYB proto-oncogene, ASXL3-miR-181c-3p-cyclin D2 (Ccnd2), and ASXL3-miR-323-3p-Ccnd2) in the PI3K-Akt pathway and six ASXL3–miRNA–mRNA networks (ASXL3-miR-206-3p-mitogen-activated protein kinase 10 (Mapk10), ASXL3-miR-34c-5p-Mapk10, ASXL3-miR-382-5p-Mapk10, ASXL3-miR-466k-Mapk10, ASXL3-miR-542-3p-Mapk10, and ASXL3-miR-708-5p-Mapk10) in the MAPK pathway, all of which may be associated with heart development.

To identify more potential ASXL3–miRNA–mRNA networks, we performed an in-depth analysis of the 11 selected miRNAs. The expression trends of six miRNAs – mmu-miR-206-3p, mmu-miR-181c-3p, mmu-miR-708-5p, mmu-miR-466d-3p, mmu-miR-485-5p, and mmu-miR-323-3p – were consistent with those reported in the literature. Furthermore, the PI3K-Akt and MAPK signaling pathways were enriched in the predictive target genes of mmu-miR-206-3p, mmu-miR-181c-3p, mmu-miR-708-5p, and mmu-miR-323-3p. The PI3K-Akt pathway, which is highly conserved, is widely noted in eukaryotes [59]. Recent studies have stated that the PI3K-Akt pathway is not only involved in cardiac ischemia-reperfusion injury, cardiac hypertrophy, and cardiac cell remodeling but also influences heart development by regulating the proliferation of cardiac precursor cells [60–63]. ASXL3, as a transcription factor, can regulate miRNAs [64,65]. The expression of miRNA is substantially reduced after interfering with ASXL3. Based on the expression trends of the above mentioned four miRNAs, we concluded that the expression of mmu-miR-323-3p is more consistent with the expected results. Therefore, we hypothesized that the ASXL3/mmu-miR-323-3p/Ccnd2 axis participates in P19 cell differentiation through relevant molecules in the PI3K-Akt pathway. Although RNA sequencing and analysis revealed multiple putative ASXL3/miRNAs/mRNAs/PI3K-Akt networks, particularly the ASXL3/mmu-miR-323-3p/Ccnd2/PI3K-Akt pathway, which may be associated with P19 cell differentiation, in vivo and in vitro studies are required to corroborate these networks. Our future goal is to conduct further in-depth studies on the interaction between ASXL3 and related pathway genes.

## 5. Conclusion

We evaluated the expression profiles of miRNA and mRNA during DMSO-induced differentiation of P19 cells. Based on DE-miRNAs and DEMs found, we used bioinformatics to identify the most promising ASXL3–miRNA–mRNA networks associated with the PI3K-Akt and MAPK signaling pathways during heart development. Our findings may shed light on the broad regulatory pathways involved in heart development, which may provide further insights into the theoretical basis for CHD therapy.

## Supplementary Material

Supplemental Material

## Data Availability

The data used to support the findings of this study are available from the corresponding author upon request.
