# Supplementary figures and images for: Identification of differential microRNAs and messenger RNAs resulting from ASXL transcriptional regulator 3 knockdown during during heart development
Source: Bioengineered. 2022 Apr 17;13(4):9948–61. doi: 10.1080/21655979.2022.2062525 (PMC9161854; doi:10.1080/21655979.2022.2062525)

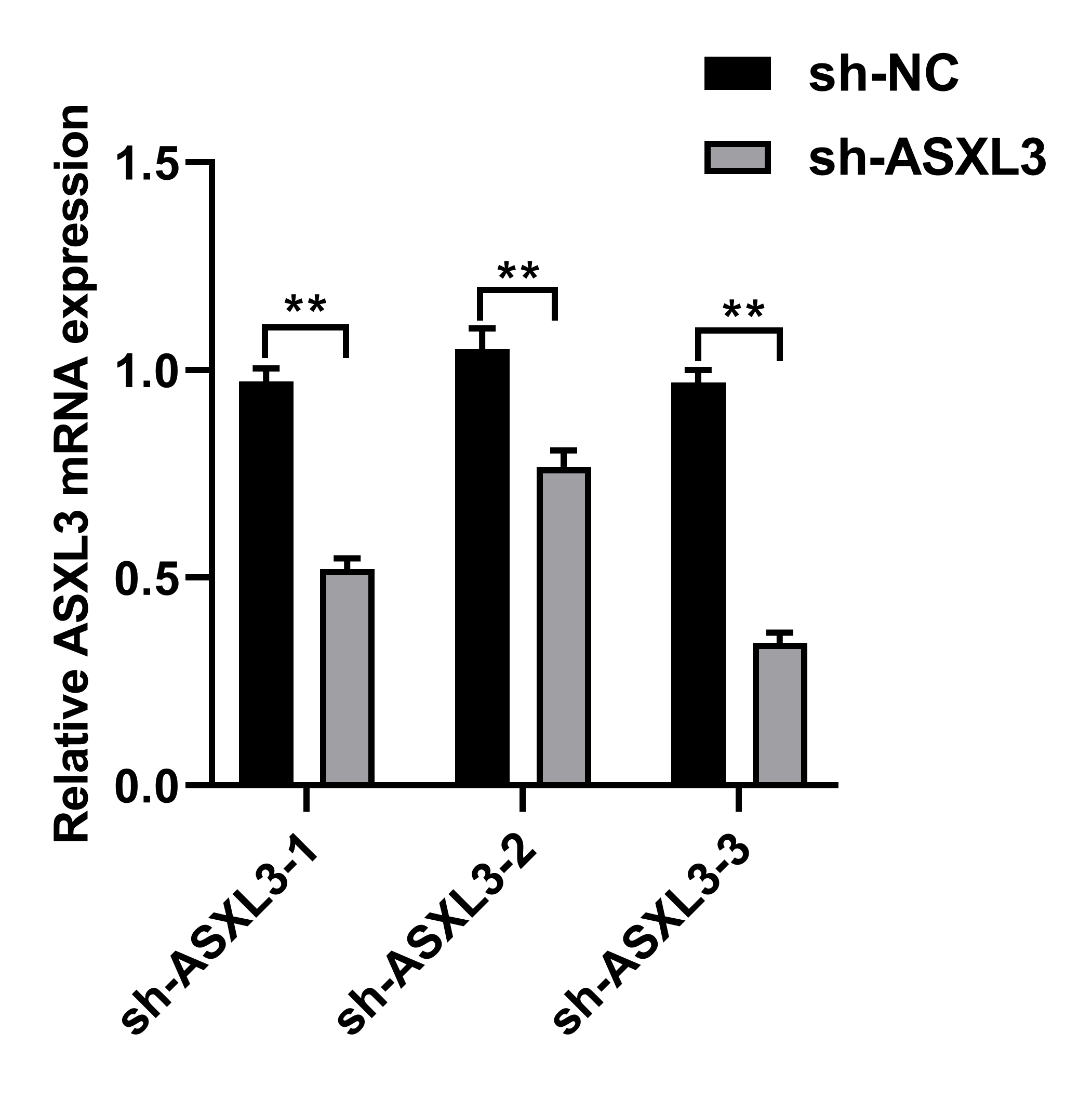

Supplement: Supplemental Material [file KBIE_A_2062525_SM4160.zip › supplementary/Supplemental Figure 1.tif]
